# Suicidal impulsivity secondary to traumatic brain injury

**DOI:** 10.1192/j.eurpsy.2024.1625

**Published:** 2024-08-27

**Authors:** J. Fernandez Logroño, C. I. Vazquez Arjona, I. Romero Mañas

**Affiliations:** UNIDAD DE SALUD MENTAL, HOSPITAL UNIVERSITARIO CLINICO SAN CECILIO, GRANADA, Spain

## Abstract

**Introduction:**

I present the case of a 58-year-old patient who developed frequent, unpredictable and prolonged suicidal impulsivity (more than 8 years of evolution) after one year of suffering a traumatic brain injury, with very serious suicide attempts in the context of very brief periods of dysthymia and no history of mental illness or any other accompanying psychopathology.

Throughout this admission, a progressive dehospitalization has also been carried out, with afternoon outings in the company of his wife or son up to a full weekend.

**Objectives:**

Shortly before, frequent “déjà vu” crises had also begun. Additional imaging tests (CT and cranial MRI) had been performed privately, which had been normal, and an EEG with sleep deprivation had been requested, but the patient had not attended.

For 8 years he had started various successive antidepressant treatments that had always been ineffective or had produced agitation, which was diagnosed as akathisia, after a week of treatment. In a single previous hospital admission, with the initial diagnosis of major depressive disorder finally ruled out, he was discharged apparently asymptomatic, and was readmitted after making three new successive serious attempts at self-lysis a week after discharge.

**Methods:**

Throughout this hospitalization (37 days), a practically invariable mental state is observed from the first day in which only rambling thoughts with very limited content stand out, with permanent and apparently credible criticism regarding previous self-harming behaviors, without appearance of new impulses or self-harming behaviors and reporting a significant decrease in the frequency and emotional impact of “déjà vu” type crises, which are now limited to the moment of waking up in the afternoon, after a brief nap, and occasionally.

**Results:**

He was discharged from the hospital with the diagnosis of post-concussive syndrome (ICD 10-F0.78.2) and remains stable for the moment (one month later) in improvement, maintaining anxiolytic and antidepressant treatment, as well as anticonvulsants, and pending continuation of the study for part of neurology.

**Conclusions:**

We think that this case shows how, within the immense etiological variety of suicidal behavior, there may be a cause conditioned exclusively by brain damage.

**Disclosure of Interest:**

None Declared

